# Editorial: Mechanisms of auditory development, maintenance, damage, and protection

**DOI:** 10.3389/fnmol.2025.1580793

**Published:** 2025-03-11

**Authors:** Ruosha Lai, Lihua Li, Su-Hua Sha

**Affiliations:** ^1^Department of Otolaryngology-Head and Neck Surgery, The Second Xiangya Hospital, Central South University, Changsha, Hunan, China; ^2^Department of Otolaryngology Head and Neck Surgery, The Second Affiliated Hospital, Jiangxi Medical College, Nanchang University, Nanchang, China; ^3^Department of Pathology and Laboratory Medicine, Medical University of South Carolina, Charleston, SC, United States

**Keywords:** auditory, development, maintenance, damage, protection

Auditory perception is fundamental to human experience, making this topic highly significant in contemporary research. Although auditory research has advanced our understanding of the mechanisms of acquired hearing loss, such as noise- and ototoxic drug-induced hearing loss and age-related hearing impairment, various challenges and limitations remain. Currently, there are no clinical pharmaceutical therapies for the prevention and treatment of acquired hearing loss, and the need for effective protective strategies underscores the urgency of further exploration of the complexities of auditory function and protection. Molecular neuroscience plays a pivotal role in elucidating the complexities of auditory development, maintenance, injury, and protection. This series of articles explores genetic and molecular neuroscientific mechanisms, cell death pathways, multi-omics analysis, clinical translation, and disease association studies to make significant progress in unraveling the mechanisms of auditory function and protection.

The study by Kang et al. used induced pluripotent stem cells (iPSCs) derived from patients with a *Tmem43* mutation to generate glia-like supporting cells (GLSs). This mutation disrupts gap junction function in GLSs, subsequently affecting auditory function. This study provides novel insights into the potential mechanisms underlying auditory neuropathy spectrum disorders (ANSD) caused by genetic mutation of the *Tmem43* gene.

The study by Zhou et al. explored the relationship between the sensation of ear fullness and the synaptic loss in the brain, using a technique called SV2A positron emission tomography (PET) to measure synaptic density in the cortex. The study shows specific brain regions where synaptic changes are associated with the presence of ear fullness, potentially providing insight into the neural mechanisms underlying this symptom.

This study by Xie et al., using the CRISPR/Cas9-generated *Myo7aa* gene knockout in zebrafish, demonstrated that *Myo7aa*, a homolog of the human Usher 1B syndrome gene *Myo7a*, influences congenital hearing and balance development in Zebrafish by modulating the Rho GTPase signaling pathway. This research suggests the pivotal role of *Myo7aa* in auditory development and identifies a potential therapeutic target for gene therapy.

The study by Wang et al. addressed the pathological mechanism underlying noise-induced hearing loss (NIHL). The authors found that noise exposure triggers apoptosis and necrosis of cochlear outer hair cells (OHCs) through the NFAT3/FasL axis. Inhibiting NFAT3 nuclear translocation or FasL expression attenuates noise-induced OHC loss, offering a novel strategy for the prevention of NIHL.

The study by Ji et al. showed that treatment with desmopressin acetate (DDAVP) significantly affects vestibular and hearing function in guinea pigs. Treatment with a traditional Chinese medicine, Zexie Decoction, attenuates DDAVP-induced damage to vestibular and auditory function in a dose-dependent manner. This study suggests that Zexie Decoction may offer a promising direction for the pharmacological intervention of acquired hearing loss.

In addition, the study by Kui et al. suggested that type 2 diabetes mellitus (T2DM) may increase the risk of developing acute suppurative otitis media (ASOM) based on univariate and multivariate Mendelian randomization (MR) analysis. This study highlights the importance of stringent glycemic control to minimize the risk of hearing impairment associated with T2DM. Additionally, two review articles by Di et al. and Jin et al. summarized sudden hearing loss and the association between hypertension and hearing loss, respectively.

Despite the substantial advances in current research, several unresolved questions and challenges remain concerning the underlying molecular mechanisms, clinical translation, and potential therapeutic intervention against auditory damage.

1. The molecular mechanisms underlying many types of hearing conditions have not been fully elucidated, particularly regarding how *Tmem43* mutations specifically impact gap junction function and the precise regulation of *Myo7aa* within the Rho GTPase signaling pathway.
2. While the Chinese medicine, Zexie Decoction, can attenuate DDAVP-induced vestibular and auditory dysfunction, it is insufficient to completely reverse the impairment, highlighting the urgent need for more effective therapeutic strategies.
3. The mechanisms governing the onset and recovery of middle ear fullness in patients with sudden hearing loss remain unclear and appear to be unrelated to the extent of recovery from hearing loss, suggesting a potentially complex neuroplastic mechanism.
4. Further investigation is required to understand how the interaction of environmental factors such as noise and metabolic diseases, along with genetic factors, influences auditory function.
5. Although existing animal models and *in-vitro* experiments can simulate certain pathological processes, they fall short of capturing the full complexity of the human auditory system, thereby limiting the clinical translation of research findings.

In conclusion, the current state of the art in this Research Topic has yielded substantial results and technological applications. However, numerous unresolved questions and research challenges remain. Future research should focus on addressing these gaps to further advance the development of auditory science and to provide innovative strategies and methods for clinical treatment.

